# Epicardial Contrast Injection to Guide Left Atrial Appendage Closure

**DOI:** 10.1016/j.hrcr.2021.04.001

**Published:** 2021-04-20

**Authors:** Thomas A. Dewland, Ayhan Yoruk, Randall J. Lee

**Affiliations:** Electrophysiology Section, Division of Cardiology, Department of Internal Medicine, University of California, San Francisco, San Francisco, California

**Keywords:** Atrial fibrillation, Epicardial, LARIAT, Ligation, Left atrial appendage closure

## Introduction

Left atrial appendage (LAA) closure can be considered in patients who are at high risk of atrial fibrillation–associated thromboembolic events but cannot tolerate long-term oral anticoagulation.^1^ A proportion of these individuals are poor candidates for endocardial LAA occlusion, owing to either complex appendage anatomy or an inability to tolerate the antiplatelet and anticoagulant regimen recommended after endocardial device implantation. In these cases, percutaneous LAA ligation may be appropriate.

Percutaneous LAA ligation uses a suture-based snare that is tightened over the epicardial surface of the proximal appendage, resulting in acute closure and subsequent necrosis of this structure.^2^ The snare is delivered over a rail system that is established by connecting 2 magnet-tipped wires positioned immediately opposite one another on the endocardial and epicardial surfaces of the appendage.

Successful and safe percutaneous LAA ligation requires placement of the endocardial magnet wire in the most distal, anterior/superior aspect of the appendage. Correct wire placement is typically confirmed by transesophageal echocardiography (TEE) and by fluoroscopic visualization using endocardial contrast injection through the transseptal sheath. In certain patients, inadequate image quality limits the utility of these techniques. We therefore describe the use of contrast injection into the epicardial space to define appendage anatomy and guide LAA ligation.

## Case report

**Figure 1:**
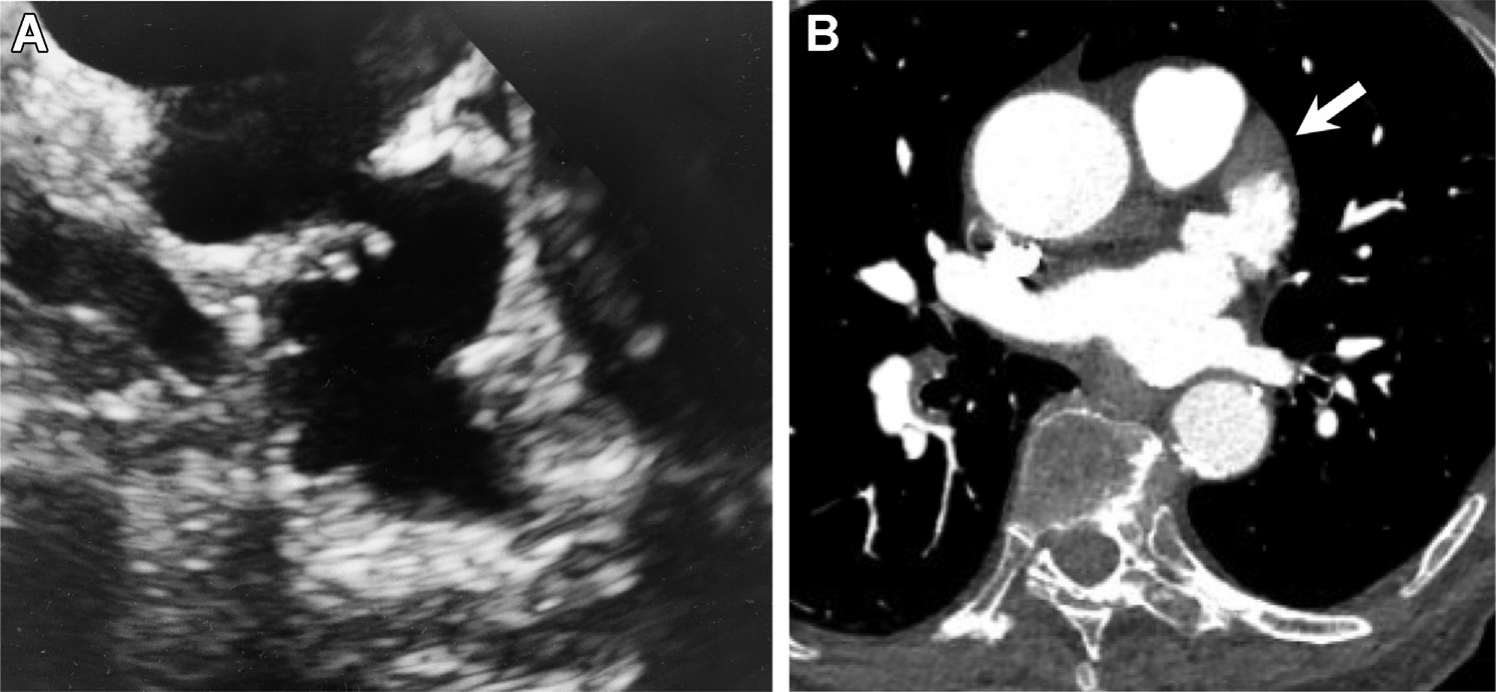
Preprocedure left atrial appendage (LAA) imaging. **A:** Transesophageal echocardiography (TEE) imaging at 135 degrees demonstrates serpiginous LAA anatomy. **B:** Similar anatomy is seen on a contrast-enhanced chest computed tomography scan. The filling defect at the LAA apex (*white arrow*) was secondary to low flow and poor contrast filling; no thrombus was seen on a subsequent intraprocedural TEE.

A 72-year-old woman with a history of persistent atrial fibrillation was treated with radiofrequency catheter ablation at an outside center. Her procedure included extensive substrate ablation that resulted in electrical isolation of the LAA. Despite maintenance of sinus rhythm and compliance with anticoagulation, the patient suffered a thromboembolic stroke 2 years after her ablation. A year later, she experienced a hemorrhagic stroke in a separate vascular territory. Watchman LAA occlusion was attempted but was aborted owing to complex appendage anatomy (Figure 1). The patient was then referred to our institution for percutaneous LAA ligation.

**Figure 2:**
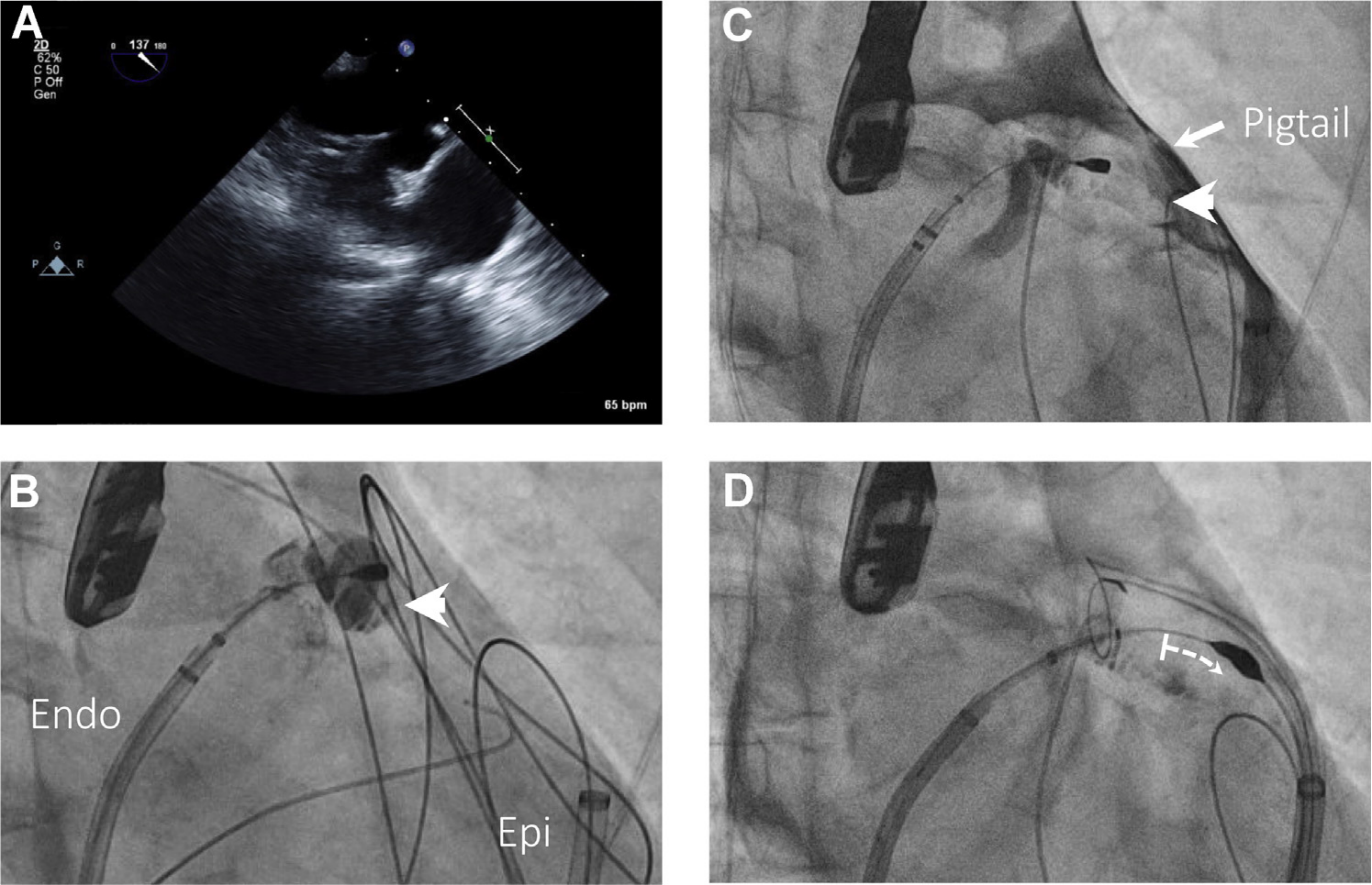
Intraprocedural imaging. **A:** Intraprocedural transesophageal echocardiography images were suboptimal and visualization of the distal appendage was difficult. **B:** The endocardial magnet is positioned in the left atrial appendage (LAA). Endocardial injection of contrast through the balloon-tipped catheter falsely suggests that the magnet is in the distal appendage (*white arrowhead*). The endocardial (Endo) and epicardial (Epi) sheaths are labeled. **C:** Epicardial contrast injection through a pigtail catheter positioned adjacent to the LAA demonstrates that the appendage extends much more anteriorly than was appreciated with endocardial contrast injection (appendage apex labeled with white arrowhead). **D:** The magnet is advanced into the distal appendage and the structure is ligated. All images were obtained in the same right anterior oblique projection.

Appendage ligation was performed with the LARIAT device (AtriCure, Mason, OH).^3^ Epicardial access was first obtained using a micropuncture technique. After transseptal puncture with an SL0 sheath, we were unable to navigate a pigtail catheter into the LAA. Hand injection of contrast through the SL0 sheath failed to sufficiently opacify the appendage. Following standard LARIAT protocol, a magnet wire was placed within a balloon-tipped catheter and advanced into the left atrium through the SL0 sheath. A slight curve placed at the end of the magnet wire allowed the magnet to be maneuvered into the LAA. Owing to suboptimal TEE image quality, it was difficult to confirm that the magnet was in the anterior/superior lobe of the LAA (Figure 2A). Injection of contrast through the balloon-tipped catheter suggested that the magnet was positioned in the distal appendage (Figure 2B).

A 6F angled pigtail catheter was inserted into the epicardial space and guided toward the appendage. Hand injection of 5 mL of iodixanol injectable contrast medium mixed with 5 mL of normal saline resulted in clear definition of the LAA border (Figure 2C, Supplemental Video) and indicated that more distal positioning of the magnet was required. After the magnet was advanced to the appendage apex, the endocardial and epicardial magnets were then connected and the LAA was ligated (Figure 2D). Complete LAA closure was confirmed by endocardial contrast injection against the closed appendage ostium through the SL0 sheath. After closure, 20 mL of saline was introduced into the epicardial space and then withdrawn to clear residual contrast. Fluoroscopy time was 11.0 minutes and total procedural time was 112 minutes.

## Discussion

We describe the use of contrast injection into the epicardial space to generate a “negative” image of the LAA to guide percutaneous ligation. This technique should be considered when TEE and endocardial contrast injection under fluoroscopy provide suboptimal imaging of the distal LAA.

Successful LAA ligation with the LARIAT technique requires the endocardial and epicardial magnets to connect at the distal aspect of the appendage. Failure to identify the LAA apex and to correctly position the endocardial magnet in the distal appendage can result in incomplete ligation and increase the risk of tear. Furthermore, blind advancement of the magnet wire without appreciation of the appendage border can increase the risk of perforation.

Epicardial contrast injection does not require additional equipment, as an angled pigtail catheter is typically used to maneuver the transseptal sheath to the LAA ostium and to fluoroscopically define appendage anatomy from an endocardial approach. In the present case, we were unable to advance the endocardial pigtail deep into the appendage, likely secondary to complex anatomy.

At the conclusion of the procedure, we elected to inject and then withdraw saline into the epicardial space in an effort to remove residual contrast. The implications of leaving contrast within this potential space are not known, although smaller amounts of contrast are routinely injected into the pericardium during epicardial access without clinical sequelae.

## Conclusion

This case illustrates the importance of incorporating multiple imaging techniques during percutaneous LAA closure. Epicardial contrast administration to precisely define appendage anatomy should be part of the LAA closure imaging armamentarium and should be considered when the appendage apex is incompletely defined by endocardial contrast injection and TEE imaging.

## Appendix Supplementary data

Supplemental Video Epicardial Contrast Injection. Epicardial contrast injection through a pigtail catheter positioned adjacent to the LAA demonstrates that the appendage extends much more anteriorly than was appreciated with endocardial contrast injection.
